# Peptide binding at the gasdermin D exosite reveals the structural basis for targeting the site

**DOI:** 10.1107/S205979832600344X

**Published:** 2026-05-13

**Authors:** Renjing Wang, Thu Ho, Aimie Ogawa, Kartika Widjaja, Zachary Brown, Song Yang, Pei Huo, Michael C. Gregory, Aman Singh, Senthil Perumal, Songnian Lin, Alan C. Cheng, Lindsay S. Garrenton, James Mu, Anthony Ogawa, Jill E. Chrencik

**Affiliations:** a Protein and Structural Chemistry, Merck & Co., Inc, 213 East Grand Avenue, South San Francisco, CA94080, USA; b Protein and Structural Chemistry, Merck & Co., Inc., 770 Sumneytown Pike, West Point, PA19486, USA; c Quantitative Biosciences, Merck & Co., Inc., 213 East Grand Avenue, South San Francisco, CA94080, USA; d Discovery Biology, Merck & Co., Inc., 213 East Grand Avenue, South San Francisco, CA94080, USA; e Discovery Chemistry, Merck & Co., Inc., 126 East Lincoln Avenue, Rahway, NJ07065, USA; f Modeling and Informatics, Merck & Co., Inc., 213 East Grand Avenue, South San Francisco, CA94080, USA; g Discovery Chemistry, Merck & Co., Inc., 213 East Grand Avenue, South San Francisco, CA94080, USA; Institute of Integrative Biology, University of Liverpool, United Kingdom

**Keywords:** gasdermin D, exosite, cyclic peptides, druggability, structure-based drug design, inhibition

## Abstract

Gasdermin D (GSDMD) has been identified as a critical component of the inflammasome, an important intracellular signaling multi-protein complex. Abnormal activation of GSDMD has been linked to a variety of inflammatory diseases, including non-alcoholic steatohepatitis, inflammatory bowel disease and COVID-19, and has been linked to potential pathogenesis of septic shock. While small molecules have been identified to inhibit N-terminal domain (NTD) pore formation and membrane translocation, further clinical application of these molecules is currently limited due to narrow specificity. Here, we report a peptide from a structure-based computational screen that selectively inhibits caspase-dependent cleavage of GSDMD. We have determined the structure of the GSDMD–peptide complex, indicating that peptide binding is facilitated by negatively charged residues from the peptide and the hydrophobic exosite of the protein, inhibiting caspase binding and activation. The experimental data suggest the potential to target the exosite for therapeutic intervention.

## Abstract 2

We describe the first structure of gasdermin D with a putative caspase inhibitor peptide analog at 1.6 Å resolution. This work suggests potential druggability of gasdermin D at the exosite by leveraging structure-based computational design with biochemical characterization.

## 1. Introduction

Pyroptosis is a caspase-dependent form of regulated cell death that results in the lysis and release of IL-1 family cytokines (IL-1β, IL-18 and IL-1α) in response to host infection and danger signals mediated by inflammasome activation (Xia, Wang *et al.*, 2019 ▸; Frank & Vince, 2019 ▸; Jackson & Theiss, 2020 ▸; He & Amer, 2014 ▸; Li *et al.*, 1995 ▸; Nunes & Souza, 2013 ▸; He *et al.*, 2015 ▸; Ouyang *et al.*, 2023 ▸). Human gasdermin D (GSDMD) is expressed in leukocytes, the respiratory system, the gastrointestinal tract, bone marrow, liver and gallbladder, and is the key executioner of pyroptosis in both canonical and noncanonical inflammasome activation (Magnani *et al.*, 2022 ▸). The canonical inflammasome activates caspase-1 in response to sensor proteins (NLR, AIM2, NAIP, NLRC4 and pyrin) that oligomerize in response to their cognate ligand, whereas the noncanonical inflammasome is activated by murine caspase-11 and its human orthologs caspase-4 and caspase-5 in response to lipopolysaccharide binding (Shi *et al.*, 2015 ▸; Xia, Hollingsworth *et al.*, 2019 ▸; Rathinam *et al.*, 2012 ▸). Distinct from pyroptosis, GSDMD also plays a vital role in NETosis, which describes the release of chromatin decorated with specific proteins that act to form neutrophil extracellular traps that capture microorganisms, activate myeloid cells and promote coagulation (Zhou *et al.*, 2023 ▸; Wang *et al.*, 2020 ▸; Liu *et al.*, 2016 ▸). Dysregulation of inflammasome activation often contributes to human diseases, particularly inflammatory diseases, and therefore inhibiting GSDMD activation may provide relief for a variety of human diseases (Rathkey *et al.*, 2018 ▸; Xu *et al.*, 2018 ▸; Khanova *et al.*, 2018 ▸; Yap *et al.*, 2020 ▸; Saeki *et al.*, 2009 ▸; Gao *et al.*, 2018 ▸; Qiu *et al.*, 2017 ▸).

GSDMD belongs to a six-member pore-forming family that includes GSDMA, GSDMB, GSDMC, GSDME and DFNB59 (Yu *et al.*, 2014 ▸; Ding *et al.*, 2016 ▸; He *et al.*, 2015 ▸; Wu *et al.*, 2009 ▸; Bergsbaken *et al.*, 2011 ▸). Except for DFNB59, all gasdermins share roughly 45% sequence homology and a conserved two-domain architecture: an N-terminal domain and a C-terminal domain with a cleavable linker connecting the two domains (Zhou *et al.*, 2023 ▸; Rogers & Alnemri, 2019 ▸). Native GSDMD adopts an autoinhibited conformation, which is released upon cleavage by caspases-1/4/5/11 at the recognition site 272-FLTD-275 in humans and 273-LLSD-276 in mice (Wang *et al.*, 2020 ▸). Upon caspase cleavage, the positive potential surface of the GSDMD N-terminal domain is released and oligomerizes into a membrane-pore structure via a charge–charge inter­action with a hydrophobic tip that inserts into the bi-lipid membrane (Aglietti *et al.*, 2016 ▸; Sborgi *et al.*, 2016 ▸; Liu *et al.*, 2016 ▸; Chen *et al.*, 2016 ▸; Russo *et al.*, 2016 ▸). Oligomerization of the human GSDMD (hGSDMD) N-terminal domain (NTD) is highly dependent on two cysteine residues (Cys38 and Cys191), which likely regulate pore formation (Liu *et al.*, 2016 ▸; Rathkey *et al.*, 2018 ▸). Xia and coworkers recently characterized the GSDMD 33-subunit pore by cryo-EM and found that it mediates mature IL-1β and IL-18 release by electrostatic filtering with extensive membrane-binding elements including a hydrophobic anchor and three positively charged patches (Xia *et al.*, 2021 ▸).

Identification of a GSDMD-specific inhibitor has proven to be exceptionally challenging. Disulfiram, a drug for treating alcohol addiction, was identified as a covalent modifier of the Cys191 and Cys192 residues corresponding to hGSDMD and mouse GSDMD (mGSDMD), respectively, by effectively inhibiting pore formation (Hu *et al.*, 2020 ▸). Another small molecule, dimethyl fumarate, reacts with the critical cysteine residues in GSDMD to form *S*-(2-succinyl)cysteine and prevents interaction with caspases. This limits the cleavage of GSDMD and subsequently the ability of GSDMD to oligomerize into the pore (Humphries *et al.*, 2020 ▸). In addition, other putative GSDMD direct binders, including LDC7559 and necrosulfonamide, have been reported, but their mechanism of action remains elusive (Sollberger *et al.*, 2018 ▸; Rathkey *et al.*, 2018 ▸; Hu *et al.*, 2020 ▸). However, a recent publication concluded that LDC7559 does not inhibit GSDMD; instead, it inhibits the phagocytic oxidative burst. A recent study has identified both inhibitory and non-inhibitory nanobodies that specifically target human GSDMD. Some of these nanobodies act as potent inhibitors of GSDMD pore formation, demonstrating nanomolar affinity. This finding provides important insights for compound-screening techniques and suggests potential applications as inhibitors of inflammasome activity (Kopp *et al.*, 2023 ▸).

To date, most reported GSDMD inhibitors target reactive cysteines and lack specificity. The recently identified exosite on GSDMD, which contains the putative caspase-recognition site, may be a promising avenue for inhibiting cleavage in a GSDMD-dependent manner by disrupting GSDMD binding of the caspase. Of interest, the caspase-1 exosite is comprised of an antiparallel β-sheet at the L1 and L2 loops that binds to the hydrophobic pocket on the GSDMD C-terminal domain (Liu *et al.*, 2020 ▸; Bar-Shavit *et al.*, 1983 ▸; Grütter *et al.*, 1990 ▸; Rydel *et al.*, 1990 ▸). In this study, we exploited this interaction by designing cyclic peptides that mimic the caspase-1 hairpin to block the exosite and prevent cleavage. We identified several peptides that selectively inhibit caspase-mediated cleavage of GSDMD. The crystal structure of a peptide (Peptide 2) complexed with the human GSDMD C-terminal domain provides insight into targeting this protein for drug-discovery initiatives.

## 2. Materials and methods

### 2.1. Peptide synthesis

Automated Fmoc solid-phase peptide synthesis was accomplished on the Liberty Blue peptide synthesizer (CEM Corp.) at a scale of 100 µmol and used Rink Amide Protide resin (0.6 mmol g^−1^ loading; CEM Corp). All amino acids were used at a concentration of 0.2 *M*, while Oxyma and diisopropylcarbodiimide (DIC) were used at 1 *M*. For each coupling step, 10 equivalents of amino acid, 10 equivalents of Oxyma and 20 equivalents of DIC were used in a single coupling format (standard microwave conditions: 90°C for 2 min) to ensure quantitative acylation at each step. The Fmoc group was removed using 20% pyrrolidine in dimethyl formamide (DMF) with 0.1 *M* Oxyma followed by extensive washing of the resin. After linear assembly and thorough washing of the resin, the peptide was then cleaved with 95% trifluoroacetic acid (TFA), 2.5% triisopropylsilane, 2.5% H_2_O. The peptide was then precipitated from diethyl ether, the residue was dissolved in dimethyl sulfoxide (DMSO) and the crude, linear peptide was purified by reverse-phase preparative HPLC (Gilson Prep HPLC, C8 column, Kromasil C8, gradient of 15–35% acetonitrile in H_2_O including 0.05% TFA). The desired fractions were pooled and lyophilized to give a white solid.

To form the disulfide bond, the purified, linear peptide was then subjected to overnight folding in a neutral phosphate-buffered saline (PBS) buffer system with 10% DMSO. In a typical reaction, purified peptide (∼50 mg) was dissolved in 9 ml 0.1 *M* PBS buffer pH 7.4 at 3 m*M* concentration, followed by the addition of 1 ml DMSO. The reaction was allowed to stir overnight, and a sample of the reaction was analyzed to confirm quantitative disulfide-bond formation. The crude reaction mixture was then purified by reverse-phase preparative HPLC (Gilson Prep HPLC, C8 column, Kromasil C8, gradient of 15–35% acetonitrile in H_2_O including 0.05% TFA). The desired fractions were pooled and lyophilized to give a white solid. Final purity and identity were confirmed by ultra-performance liquid chromatography–mass spectrometry (UPLC-MS).

### 2.2. Peptide HPLC purification

Preparative reversed-phase HPLC of crude peptides was performed with a preparative Gilson system using a C8 (Reprosil, 200 Å, 5 µm) reversed-phase column. Appropriate linear gradients of increasing concentration of acetonitrile in water with 0.1% TFA were used at a flow rate of 85 ml min^−1^ (typical wide gradients would be 20–80%, whereas more focused gradients would be 40–60%). Fractions containing the desired product (as shown by analytical LC-MS) were combined and lyophilized.

### 2.3. LC-MS

UPLC-MS was performed on a Waters Acquity UPLC 393 system equipped with the following analytical column: Waters BEH130 C4 (2.1 × 394 100 mm, 1.7 µm). Chromatographic runs were typically performed as 2 or 5 min runs as required at a temperature of 45°C, applying linear gradients of aceto­nitrile in H_2_O (0.1% TFA was included as a mobile-phase modifier; typical wide gradients for analysis would 10–90% water in acetonitrile) with a flow rate of 0.4 ml min^−1^ and UV detection at 215 nm. Mass analysis was performed on a Waters SQ2 detector with electrospray ionization in positive ion-detection mode with a scan range of 500–1900 (mass-to-charge ratio).

### 2.4. Expression and purification of GSDMD

For fluorescence resonance energy transfer (FRET) experiments GSDMD was designed with N-terminal FLAG and C-terminal BirA sequences to enable recognition by labeled anti-FLAG and streptavidin, respectively. Expression was enhanced via TEV-cleavable N-terminal maltose-binding protein such that the final construct was pFastBac1/MG-8×His-MBP-TEV-FLAG-GSDMD (2–484)-Avi. The protein was expressed in Sf21 cells in Sf-900 II SFM medium at 26°C for 48 h, with the pellets harvested by centrifugation. The cell paste was resuspended in buffer *A* (30 m*M* Tris pH 8.0, 300 m*M* NaCl, 5% glycerol, 2 m*M* β-mercaptoethanol) and lysed by homogenization. The lysate was clarified via ultracentrifugation at 75 000*g* and GSDMD was immobilized on Ni-Sepharose HP beads (Cytiva), washed with buffer *A* and eluted with buffer *A* supplemented with 150 m*M* imidazole. Next, protein was captured on amylose resin (New England Biolabs), washed with buffer *A* and eluted with buffer *A* supplemented with 10 m*M* maltose. Partially purified GSDMD was biotinylated by BirA protein (Avidity LLC) according to the manufacturer’s instructions and liberated from MBP by the addition of TEV protease during dialysis against buffer *A*. TEV and free His-MBP were removed via subtractive IMAC in buffer *A*, with the GSDMD flowthrough further purified on a HiLoad 26/60 Superdex 200 (Cytiva) column equilibrated in buffer *A*. Caspase-1 P20 (123–296) and P10 (314–404) D381E were subcloned into pET-28a and expressed individually in *Escherichia coli* BL21 (DE3) RIPL cells (Agilent) in LB medium. Expression was induced by the addition of isopropyl β-d-1-thiogalactopyranoside (IPTG) to 0.2 m*M* and the cells were harvested by centrifugation after 3 h at 37°C. The cells were resuspended in 50 m*M* sodium phosphate pH 7.4 and lysed by sonication. The lysate was clarified by centrifugation at 30 000*g* and the inclusion bodies were washed twice with 50 m*M* Tris pH 8.0, 300 m*M* NaCl, 1 *M* guanidinium–HCl, 0.1%(*v*/*v*) Anapoe X-100 and twice with 50 m*M* Tris pH 8.0, 300 m*M* NaCl, 1 *M* guanidinium–HCl. The inclusion bodies were solubilized in 6 *M* guanidinium–HCl and the P10 and P20 solutions were mixed and subsequently refolded by rapid dilution in 50 m*M* HEPES pH 8.5, 100 m*M* NaCl, 10 m*M* DTT, 10%(*w*/*v*) sucrose, 0.1% CHAPS. Refolded caspase-1 was dialyzed against 50 m*M* sodium acetate pH 5.9, 50 m*M* NaCl, 5% glycerol, 4 m*M* DTT and purified on a HiLoad 26/60 Superdex 75 column (Cytiva) equilibrated in 25 m*M* HEPES pH 8.0, 2 m*M* DTT.

For crystallization studies, human GSDMD-CTD was cloned, expressed and purified as described previously (Anton *et al.*, 2017 ▸). In brief, hGSDMD (276–484) was subcloned into the vector pET-28b(+) with an N-terminal 6×His-MBP2 tag followed by a TEV cleavage site. *E. coli* BL21 CodonPlus (DE3) RIPL competent cells were transformed and cultured in Terrific Broth supplemented with kanamycin and chloramphenicol. The culture was induced with 0.20 m*M* IPTG at OD_600_ = 0.60 at 18°C. The cells were harvested after 18 h and the cell paste was lysed with 20 m*M* Tris pH 7.6, 150 m*M* NaCl, 10 m*M* KCl, 0.5 m*M* TCEP, EDTA-free protease inhibitor tablets, nuclease (100 U ml^−1^) and lysozyme (50 kU g^−1^) using a manual homogenizer followed by sonication. The lysate supernatant was loaded onto a 10 ml HisTrap column (Cytiva), washed with 20 m*M* Tris pH 7.6, 150 m*M* NaCl, 0.5 m*M* TCEP and eluted over an imidazole gradient. Fractions were collected and pooled for overnight TEV protease cleavage dialyzed with 20 m*M* Tris pH 7.6, 100 m*M* NaCl, 1 m*M* TCEP. The cleaved protein mixture was further purified by a subtractive IMAC step followed by gel filtration on a Superdex 75 16/60 column (Cytiva) with a final buffer consisting of 20 m*M* Tris pH 7.6, 50 m*M* NaCl, 0.5 m*M* TCEP. The GSDMD–peptide complex was prepared by adding 20× peptide to 1 mg ml^−1^ GSDMD protein and was concentrated to around 10 mg ml^−1^ in 20 m*M* Tris pH 7.6, 50 m*M* NaCl, 0.5 m*M* TCEP.

### 2.5. SEC-MALS

Human GSDMD (276–484) was incubated with a twofold molar excess of Peptide 2 at 4°C prior to injection onto SEC-MALS for comparison with its apo form. The proteins were injected onto a Superdex 200 10/300 column (Cytiva) with gel-filtration buffer consisting of 20 m*M* Tris pH 7.5, 50 m*M* NaCl, 0.5 m*M* TCEP flowing at 0.4 ml min^−1^ (Agililent Infinity system). Multi-angle light scattering was performed using the Wyatt Dawn and Optilab platforms. Data were analyzed using the Wyatt *ASTRA* software.

### 2.6. Crystallization, X-ray diffraction and structure determination

Screening of crystallization conditions was performed using commercial kits. Crystals were optimized and grown by hanging-drop vapor diffusion at 16°C using a reservoir solution consisting of 0.1 *M* HEPES pH 7.5, 1.25 *M* sodium citrate dihydrate, 20 m*M* TCEP. Crystals were harvested in crystallization buffer, serially transferred into the same buffer supplemented with 8, 16 and 24%(*v*/*v*) glycerol and flash-cooled in liquid nitrogen. All data were collected at −170°C on the IMCA-CAT Sector 17 beamline of the Advanced Photon Source and were processed with *autoPROC* (version 1.1.7) from Global Phasing Ltd. The apo structure of the C-terminal domain of hGSDMD (PDB entry 5nh1) was used as the search model in molecular-replacement calculations with *Phaser* (McCoy *et al.*, 2007 ▸). Manual model building and refinement were performed with *Coot* (Emsley & Cowtan, 2004 ▸) and *autoBUSTER* (*BUSTER* v.2.11.8; Global Phasing Ltd), respectively. The crystal structures were validated using *MolProbity* from *Phenix* (Adams *et al.*, 2010 ▸; Chen *et al.*, 2010 ▸) which demonstrated *MolProbity* scores of >2.0 at the 100th percentile (best) among structures of similar resolutions. Figures were prepared using *PyMOL* v.2.1.1 (Schrödinger) and *MOE* 2022.02 (Chemical Computing Group).

### 2.7. GSDMD-cleavage TR-FRET assay

Caspase-1-mediated cleavage of GSDMD was measured using time-resolved FRET (TR-FRET) in a buffer consisting of 50 m*M* HEPES pH 7.6, 150 m*M* NaCl, 0.05% Tween-20, 1% DMSO. Briefly, cleavage reactions were initiated by adding 150 n*M* recombinant caspase-1 to duplicate wells containing 100 n*M* recombinant biotinylated FLAG-GSDMD-Avi-tagged protein that was pre-incubated for 30 min at room temperature (RT) with a dilution series of peptides solubilized in DMSO. Each replicate reaction was then stopped after a 5 or 120 min incubation at RT by addition of the pan-caspase inhibitor Z-VAD-FMK (R&D Systems) at 10 µ*M*. After 10 min, the TR-FRET detection reagents were added as a 1:1 mixture of anti-FLAG M2-Eu cryptate mAb and Streptavidin-XL665 prepared in PPI Europium detection buffer (Revvity) to final concentrations of 0.074 and 2.45 ng µl^−1^, respectively, per the manufacturer’s instructions. After 30 min, the TR-FRET signals were recorded on an Envision plate reader (Revvity) with excitation at 340 nm and emission at 615 and 665 nm. TR-FRET signals were expressed as the ratio of fluorescence intensity at 665 nm to fluorescence intensity at 615 nm multiplied by 10^4^. The TR-FRET signals from the duplicate reactions stopped after 5 and 120 min were used to calculate the rate of cleavage. The derived reaction rates were then normalized using the formula percentage inhibition = 100 − [(*R*_raw_ − *C*_min_)/(*C*_max_ − *C*_min_)] × 100, where *R*_raw_ is the rate of cleavage for the test sample, *C*_min_ represents the minimal cleavage rate where caspase-1 is fully inhibited by 100 µ*M*Z-VAD-FMK and *C*_max_ represents the maximal cleavage rate with DMSO. The EC_50_ for each peptide was then determined using a four-parameter logistic nonlinear regression model (*Y* = bottom + (top − bottom)/{1 + 10^[log(EC_50_) − *X*] × Hill slope^}) of a semi-logarithmic plot of percentage inhibition versus compound concentration.

### 2.8. Caspase-1 activity assay

Caspase-1 activity was measured using a fluorogenic substrate in buffer consisting of 50 m*M* HEPES pH 7.6, 150 m*M* NaCl, 0.05% Tween-20, 1% DMSO. Briefly, reactions were initiated by the addition of 60 µ*M* Ac-WEHD-AFC substrate (Cayman Chemical) to 75 n*M* recombinant caspase-1 that had been pre-incubated for 30 min at RT with a dilution series of peptides solubilized in DMSO. After an additional 30 min incubation, free AFC was measured on an Envision plate reader (Revvity) with excitation at 405 nm and emission at 510 nm. The fluorescence values were then normalized using the formula percentage inhibition = 100 − [(*S*_raw_ − *A*_min_)/(*A*_max_ − *A*_min_)] × 100, where *S*_raw_ is the fluorescence value for the test sample, *A*_min_ represents the minimal enzyme activity with full inhibition by 100 µ*M* Z-VAD-FMK and *A*_max_ represents the maximal enzyme activity with DMSO. The EC_50_ for each peptide was then determined using a four-parameter logistic nonlinear regression model (*Y* = bottom + (top − bottom) /{1 + 10^[log(EC_50_) − *X*] × Hill slope^}) of a semi-logarithmic plot of percentage inhibition versus compound concentration.

## 3. Results

### 3.1. Structure-based design of a peptide inhibitor of GSDMD cleavage

**Figure 1:**
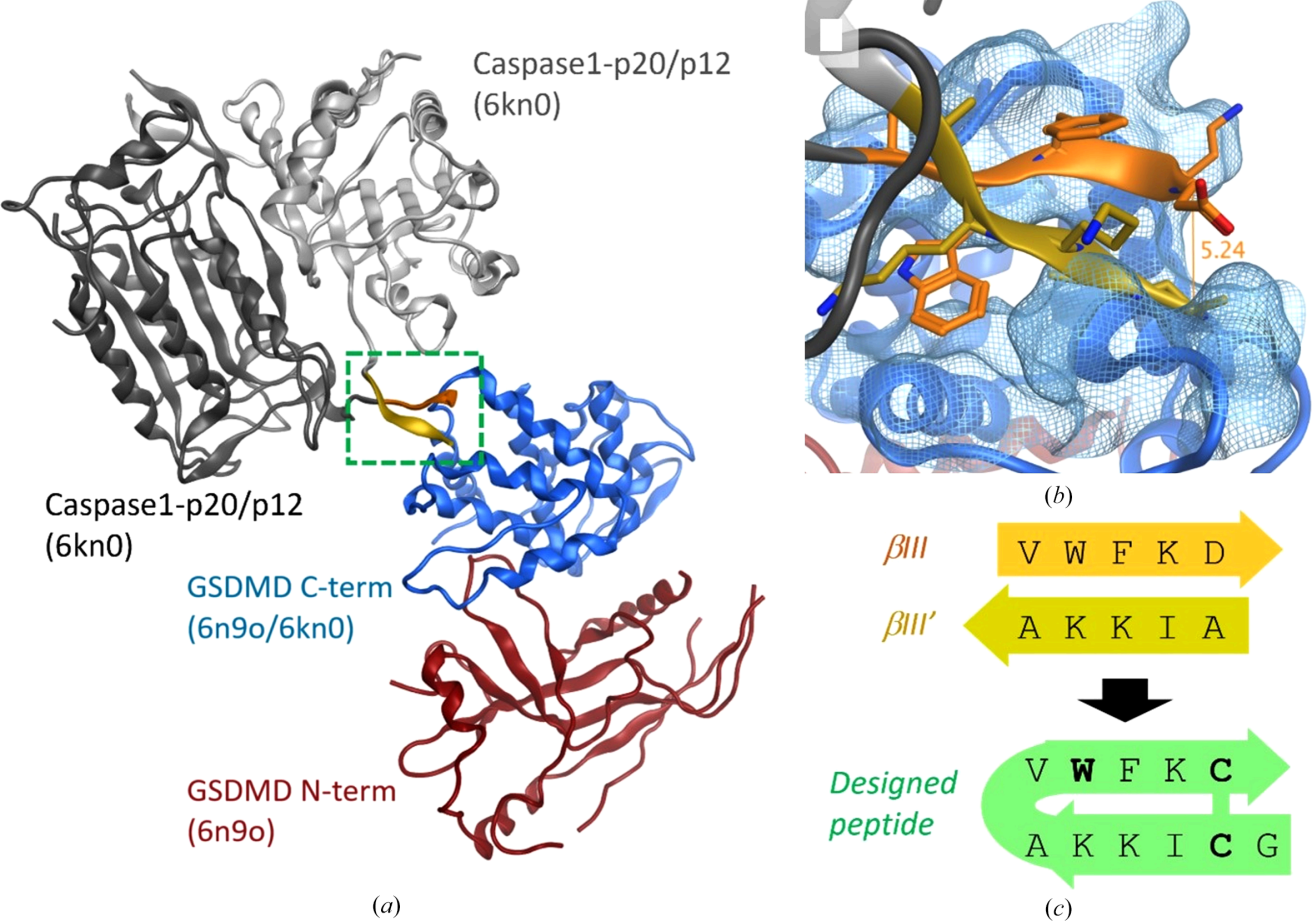
Structure-based design of a cyclic GSDMD peptide to inhibit cleavage and pore formation. (*a*) Using available co-crystal structures of caspase-1 and GSDMD (Wang *et al.*, 2020 ▸; Liu *et al.*, 2020 ▸), we postulated that a cyclic peptide could bind at the caspase-1 βIII dimer binding site highlighted by the green dashed box. (*b*) The two caspase-1 βIII peptide-binding segments with side chains rendered as sticks. (*c*) Using structure-based design and computation, a set of single cyclic peptides were designed. Residues in bold were modified based on structure-based design and computational residue scanning.

To confirm that GSDMD-NTD does not interfere with the interaction between the caspase-1 P20/P12 subunits and GSDMD-CTD, available GSDMD crystal structures were superimposed. This analysis revealed that the βIII strands of the caspase-1 dimer form an antiparallel pair of β-strands that interact with GSDMD-CTD, as shown in Fig. 1 ▸(*a*). We postulated that the βIII/βIII′ strands were necessary and sufficient for binding at the exosite and that a peptide binding at the region may prevent caspase-1 binding and therefore GSDMD cleavage and GSDMD-mediated pyroptosis. Careful inspection of the βIII/βIII′ strand region in the co-crystal structure with PDB code 6kn0 (Fig. 1 ▸*b*) resulted in the design of a compound (Peptide 1), depicted in Fig. 1 ▸(*c*) and Table 1 ▸, where two point mutations, D297C and A318C, enable a pair of cysteine residues to form a disulfide bond that stabilizes the β-hairpin conformation. Expanding on the direct sequence mimetic Peptide 1, we sought to capitalize on potentially favorable interactions with the GSDMD exosite. To this end, we performed residue scanning using *MOE* 2019.0102 (Chemical Computing Group) with an internal non-natural amino-acid collection and using a small-molecule force field, MMFF94x, with Born solvation. The residue scanning identified that a 6-chlorotryptophan (6CLW) replacement for Trp294 and a G1F point mutant were predicted to improve affinity. The four designed peptides are shown in Table 1 ▸ and the quality-control mass-spectrometry data are shown in Supplementary Fig. S1.

### 3.2. Exosite peptides selectively inhibit caspase-1-mediated cleavage of GSDMD *in vitro*

**Figure 2:**
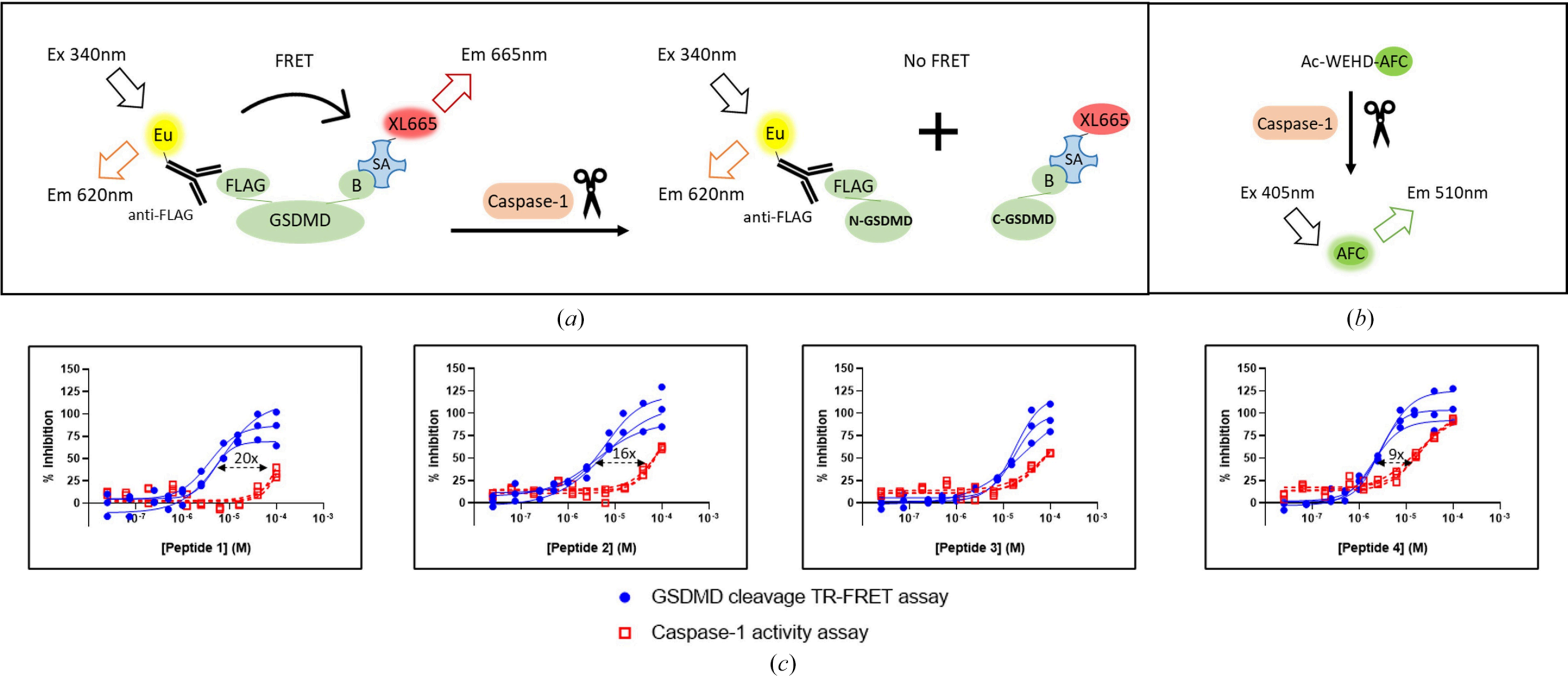
Biochemical characterization of exosite peptides in GSDMD-cleavage TR-FRET and caspase-1 activity assays. (*a*) Schematic of the GSDMD-cleavage TR-FRET assay. Two fluorophores are used for detection and quantitative analysis of FRET signals: europium cryptate conjugated to anti-FLAG M2 antibody serves as the donor with a maximum emission peak at 620 nm and XL665 conjugated to streptavidin serves as the acceptor with a maximum excitation at 615 nm. An intact N-terminally FLAG-tagged and C-terminally biotinylated full-length GSDMD protein results in emission of the acceptor XL665 at 665 nm following excitation by energy transfer from the donor Eu and cleavage by caspase-1 leads to a decrease in the emission of the acceptor at 665 nm. (*b*) Schematic of the caspase-1 activity assay. A fluorogenic tetrapeptide (Ac-WEHD-AFC) was used for quantitative analysis of caspase-1 activity. The AFC fluorophore is quenched in the intact peptide, whereas cleavage by caspase-1 results in increased free AFC and emission at 510 nm. (*c*) GSDMD-cleavage TR-FRET assays (blue circles) and caspase-1 assays (red squares) were performed in the presence of increasing concentrations of peptides in three independent experiments (*n* = 3). Selective inhibition of GSDMD cleavage compared with nonspecific inhibition of caspase-1 is indicated. Abbreviations: Eu, europium cryptate; SA, streptavidin; B, biotin

To assess the ability of the peptides to inhibit GSDMD cleavage by caspase-1, a time-resolved fluorescence resonance energy transfer (TR-FRET) assay was developed using recombinant dual-tagged GSDMD and caspase-1 proteins (Fig. 2 ▸). The cleavage of GSDMD was measured as the rate of decrease in FRET signal in the presence of increasing concentrations of peptides. Consistent with our modeling predictions, all four peptides effectively inhibited the cleavage of GSDMD. Peptide 1, Peptide 2 and Peptide 4 blocked cleavage with similar EC_50_ values of 4.8, 5.3 and 2.5 µ*M*, respectively, whereas Peptide 3 was significantly weaker with an EC_50_ value of 19 µ*M* (Table 2 ▸ and Fig. 2 ▸*c*). Interestingly, when the GSDMD-cleavage TR-FRET assay was run in the presence of the reducing agent DTT, the peptides showed no detectable inhibition (Supplementary Fig. S2), suggesting that the β-hairpin conformation is critical for the peptides to occupy the GSDMD exosite and inhibit cleavage.

As mentioned above, most currently available GSDMD inhibitors lack specificity. To determine whether the activity of the peptides was specific to blocking GSDMD cleavage as opposed to generally inhibiting caspase-1 activity, the peptides were also tested in a caspase-1 activity assay using a fluorogenic caspase substrate (Fig. 2 ▸*b*). Peptides 1–3 were very weak inhibitors of caspase-1, with EC_50_ values of >100, 83 and 93 µ*M*, respectively, whereas Peptide 4 was more potent, with an EC_50_ value of 22 µ*M* (Table 2 ▸ and Fig. 2 ▸*c*). Comparing the activity of peptides in both assays, Peptide 1 and Peptide 2 were the most selective inhibition of GSDMD cleavage, with over 20-fold and 16-fold selectivity, respectively.

### 3.3. Structure of the GSDMD–Peptide 2 complex

**Figure 3:**
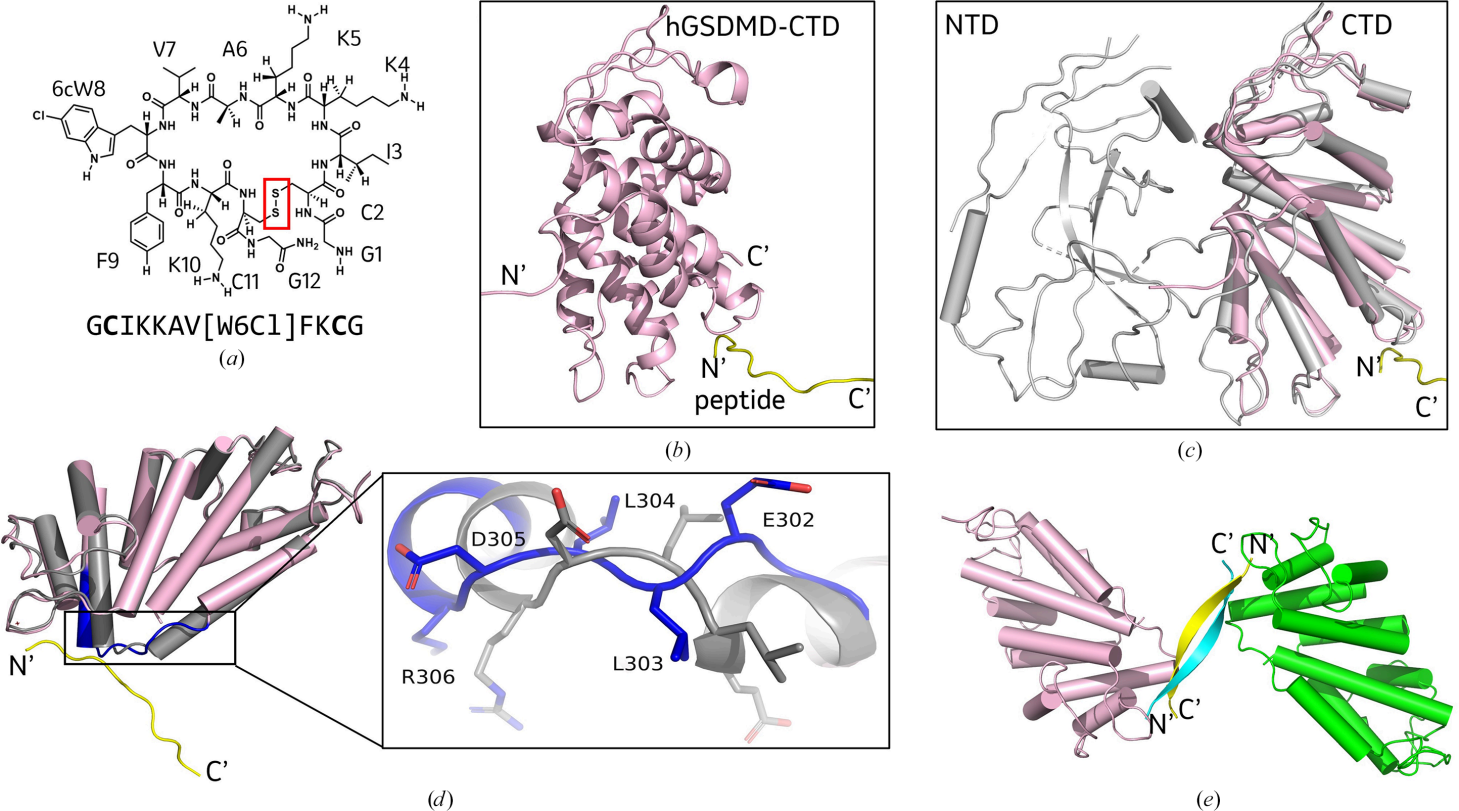
Structure of hGSDMD-CTD–Peptide 2 and the conformational changes upon peptide binding. (*a*) Chemical structure of the cyclic Peptide 2. The S—S bond for peptide cyclization is highlighted in the red box. (*b*) Global structure of the hGSDMD–Peptide 2 complex. hGSDMD-CTD is colored pink and the peptide is in yellow. (*c*) Superposition of the hGSDMD-CTD–Peptide 2 complex structure (pink/yellow) with apo full-length hGSDMD (gray, PDB entry 6n9o). The N-terminus and C-terminus are labeled N′ and C′, respectively. 1271 atoms were aligned with an r.m.s.d. of 1.336 Å. (*d*) Superposition of the hGSDMD-CTD–Peptide 2 complex structure (pink) with apo hGSDMD-CTD (gray, PDB entry 5n1h). The area of observed conformational change for the hGSDMD structure is highlighted in the blue box. α-Helices are shown as cylinders. A close-up view of the hGSDMD conformational change at the interface. The hGSDMD-CTD–Peptide 2 complex is shown in blue and apo hGSDMD-CTD is in gray. (*e*) Overall structure of two adjacent hGSDMD-CTD–Peptide 2 complex molecules in the asymmetric unit displaying the binding interface of the two linearized peptides. The two hGSDMD-CTD molecules are colored pink and green, and the peptides are displayed in yellow and cyan, respectively.

While all four peptides were tested for co-crystallization with hGSDMD-CTD, only Peptide 2 formed crystals, which diffracted to 1.6 Å resolution (data in Table 3 ▸). hGSDMD-CTD forms a compact α-helical fold consisting of ten α-helices with peptide bound on the surface (Figs. 3 ▸*a* and 3 ▸*b*). The overall hGSDMD-CTD structure aligns well with the previously reported full-length apo hGSDMD structure (PDB entry 6n9o), with a root-mean-square deviation (r.m.s.d.) of 1.336 Å for 1271 aligned main-chain atoms (residues 276–484; Fig. 3 ▸*c*; Wang *et al.*, 2020 ▸). Unsurprisingly, there are no substantial global structural changes for the individual α-helices on comparing the apo hGSDMD-CTD structure (PDB entry 5n1h) with our hGSDMD-CTD–Peptide 2 complex structure, except near the peptide-binding site (Fig. 3 ▸*d*; Anton *et al.*, 2017 ▸; Wang *et al.*, 2020 ▸; Liu *et al.*, 2020 ▸). The hGSDMD exosite loop (residue 302–306) is flipped, which rearranges residues for peptide binding, and indicates that this loop region is flexible and can adapt to bind compounds with favorable features. Although we intended to capture the structure of hGSDMD bound to the cyclic peptide, serendipitously the peptide linearized because of the presence of a reducing agent (10 m*M* TCEP) in the crystal-growth condition. As a result, two linearized peptides form a β-sheet between the two hGSDMD-CTD–peptide molecules in the crystallographic asymmetric unit (Fig. 3 ▸*e* and Supplementary Fig. S3). Similar crystal packing has been reported for the GSDMD–caspase complex, in which caspase P20 forms β-sheets with caspase P10 from the other molecule in the asymmetric unit (Liu *et al.*, 2020 ▸; Wang *et al.*, 2020 ▸). The N-terminal end of one peptide and the C-terminal end of the second peptide bind each exosite pocket in each GSDMD molecule, holding the proteins together as a dimer. However, the hGSDMD–Peptide 2 complex does not appear to be a dimer in solution based on our SEC-MALS data, which indicate that the protein is a monomer in solution (Supplementary Fig. S4). hGSDMD-CTD has a total solvent-accessible surface are of 2200 Å^2^ and the peptide-binding surface area covers over 670 Å^2^ (Lee & Richards, 1971 ▸). The hairpin formed between the two linearized peptides fully occupies the binding pocket, which we will discuss in detail below.

### 3.4. Peptide 2 binds at the hydrophobic exosite

**Figure 4:**
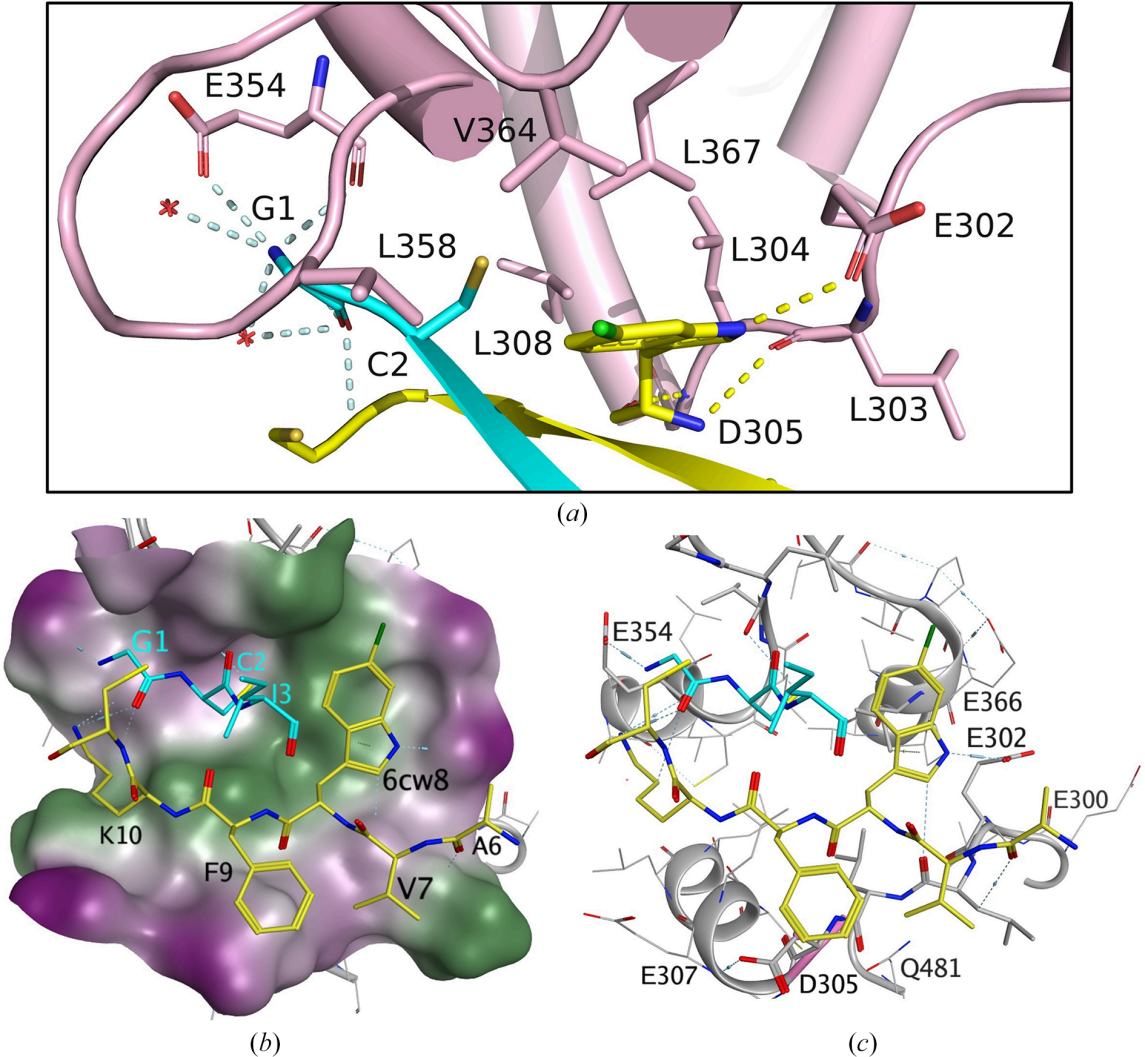
hGSDMD-CTD–Peptide 2 binding interface and surface. (*a*) A close-up view of the hGSDMD interface at the exosite. The hydrogen bonds between GSDMD and Peptide 2 are shown as yellow dotted lines and hydrogen bonds to the other peptide molecule in the asymmetric unit are shown as gray dotted lines. Water molecules are shown as red crosses. (*b*) The surface of the hGSDMD exosite binding pocket colored by lipophilicity. The key residues of Peptide 2 that are involved in the interaction with hGSDMD are shown in stick representation. Green and purple colors indicate hydrophobic and hydrophilic surfaces, respectively. (*c*) The hydrophobic binding pocket is surrounded by acidic residues. hGSDMD is colored gray and the peptide key interaction residues are colored yellow and cyan, respectively. The blue cylinders with dashed lines represent hydrogen bonds. Longer cylinders indicate stronger hydrogen bonds.

Our internal design and assay results indicate that the β-sheet of the peptide occupies the exosite of hGSDMD, which was previously identified as the caspase-recruitment module. As a result of the peptide-binding event, local rearrangements in this area are observed. In particular, residue Glu302 is flipped nearly 180° from what was observed in the apo structure, leading to a rearrangement of the nearby residues Leu303, Leu304, Asp305 and Arg306 (Fig. 3 ▸*d*, Supplementary Fig. S5). The non-native residue 6CLW8 from one linearized peptide packs against the hydrophobic exosite consisting of residues Leu303, Leu304, Leu308, Leu358, Val364 and Leu367 (Fig. 4 ▸*a*). These six hydrophobic residues are conserved in GSDMD species, but not in other gasdermin orthologs. In addition to the stacking observed between 6CLW8 and the hydrophobic residues, Peptide 2 residue 6CLW8 also forms a side-chain hydrogen bond with hGSDMD residue Glu302 with a distance of 1.83 Å and main-chain hydrogen bonds with the hGSDMD residues Leu303 with a distance of 2.15 Å and Asp305 with a distance of 1.85 Å. The GSDMD exosite is characterized by a hydrophobic groove that is surrounded by negatively charged residues, including Glu300, Glu302, Asp305, Glu307, Glu354, Glu366 and Gln481, which are all conserved in human and mouse (Figs. 4 ▸*b* and 4 ▸*c*). Peptide 2 residues Val7, 6CLW8, Phe9 and Lys10 all occupy the hydrophobic groove of the GSDMD exosite, while the residues surrounding Glu302 form hydrogen bonds to 6CLW of Peptide 2. To fully occupy the exosite, the second molecule in the asymmetric forms a β-sheet with Peptide 2 and partially occupies the exosite (Figs. 4 ▸*a* and 4 ▸*b*). Gly1 forms hydrogen bonds to the hGSDMD main-chain residue Glu354 with a distance of 2.39 Å and to two adjacent water molecules. Cys2 also forms a polar interaction with the main chain of Val357. It is important to note that our well resolved electron density for residues Glu302, Asp305 and Glu366 guided the assignment of side chains without ambiguity as a result of the high resolution of this structure, validating our findings (Supplementary Fig. S6). In order to further validate these interactions, we used energy minimization to computationally predict polar interactions between Peptide 2 and hGSDMD. Our results indicate that interactions between Peptide 2 and residues Asp305, Glu307, Glu366 and Glu354 are highly scored computationally (Supplementary Fig. S7). In summary, our data suggested that the hydrophobic groove along with the surrounding negatively charged surface are the two key factors that allow access for peptides or other modalities to occupy the exosite pocket.

### 3.5. The hGSDMD-CTD–Peptide 2 interface is similar to that observed for the GSDMD–caspase interaction

**Figure 5:**
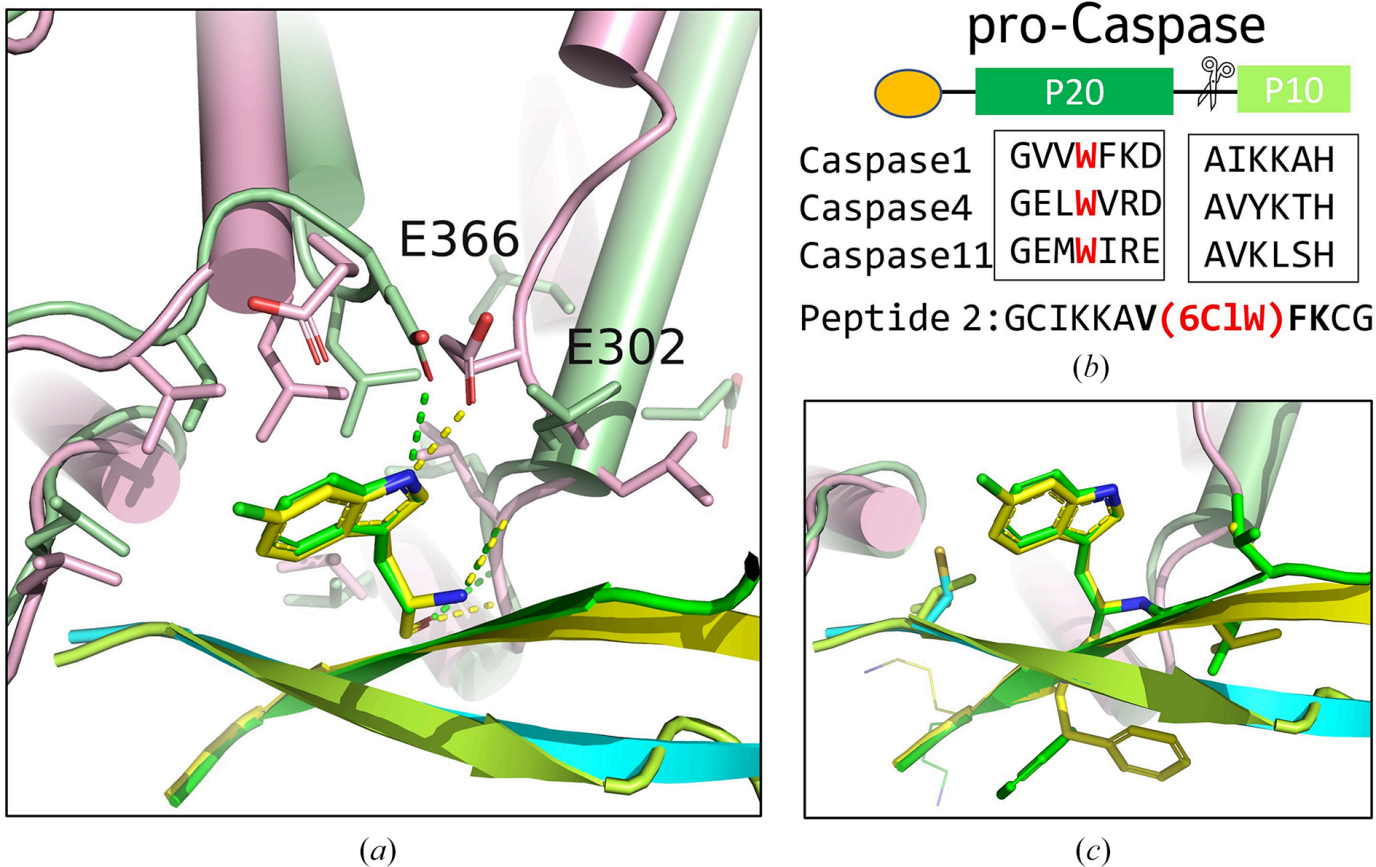
Structure comparison of hGSDMD-CTD–Peptide 2 with hGSDMD-CTD–caspase-1. (*a*) Superposition of the hGSDMD-CTD–Peptide 2 exosite (hGSDMD in pink and Peptide 2 in yellow and cyan) onto the hGSDMD-CTD–caspase-1(P20/P10) complex structure (PDB entry 6kn0) in green. The hydrogen bonds are colored yellow for peptide and green for caspase-1. (*b*) Partial sequence alignment of caspase-1, caspase-4 and caspase-11 involved in forming β-sheets and the Peptide 2 sequence. (*c*) Superposition of the hGSDMD-CTD interface of Peptide 2 onto P20/P10 with the same color scheme as in (*a*)

To compare the hGSDMD–substrate interface between the peptide and caspase structures, we aligned our structure with the hGSDMD-CTD–caspase-1 structure (PDB entry 6kn0; Fig. 5 ▸*a*). The superposition indicated no significant conformational changes for the overall structure, with an r.m.s.d. of 0.984 Å for 1173 aligned main-chain atoms (residues 276–484). Of note, however, is the key interaction of residue Trp294 of caspase-1, which is notably different between the caspase-1 structure (PDB entry 6kn0) and Peptide 2 structure (Fig. 5 ▸*a*). In the hGSDMD-CTD–caspase-1 complex, hGSDMD residue Glu366 forms a side-chain hydrogen bond to Trp294 from caspase-1, while we observe a hydrogen bond between 6CLW8 and Glu302 in our hGSDMD-CTD–peptide structure. As a result of the local conformational changes caused by the peptide, the six conserved hydrophobic residues at the exosite also shift, although the overall hydrophobic binding pocket remains intact for substrate binding (Fig. 5 ▸*a*). The key residues of caspase P20 and P10 involved in binding hGSDMD are conserved in different caspases (Fig. 5 ▸*b*). Similarly, the linearized peptides from Peptide 2 pack into β-sheets to mimic the β-sheets of P20 and P10, and the key residues from Peptide 2 involved in interactions with hGSDMD-CTD are conserved (Fig. 5 ▸*c*). Together with 6CLW8, residues Val7, Phe9 and Lys10 (highlighted in bold in Fig. 5 ▸*b*) stack against the hydrophobic pocket and occupy the conserved exosite binding interface. In conclusion, our data suggest that the GSDMD exosite possesses flexibility to accommodate different substrates, although the fundamental hydrophobic feature of the pocket is conserved.

## 4. Discussion

The recognition and cleavage of hGSDMD by inflammatory caspases are critical molecular events to initiate downstream pyroptosis in both canonical and noncanonical inflammasome signaling pathways (Shi *et al.*, 2015 ▸; He *et al.*, 2015 ▸). The molecular insight into GSDMD activation has been explored broadly, and structural studies have provided a detailed molecular basis for the hGSDMD activation mechanism. Blocking pore formation and caspase binding have attracted intense interest; however, no hGSDMD-specific inhibitors have been reported so far in the clinic. It is now clear that dysregulated hGSDMD activation can contribute to the pathology of numerous diseases, and hGSDMD has emerged as a promising therapeutic target for drug-discovery initiatives (Magnani *et al.*, 2022 ▸). Of particular interest is inhibiting hGSDMD as a mechanism to attenuate all inflammasome pathways instead of just one specific inflammasome, as is the case for NLRP3 inhibitors.

In our study, we report the first co-crystal structure of a peptide that inhibits hGSDMD cleavage. The exosite of hGSDMD may play a critical role in the initial enzyme–substrate association, and mutagenesis targeting this pocket is correlated with a reduction in the caspase cleavage of hGSDMD (Wang *et al.*, 2020 ▸; Liu *et al.*, 2020 ▸). Our structure-based cyclic peptide design (caspase hairpin-mimicking peptides) confirms that Peptide 1 and Peptide 2 inhibit casapse-1-mediated GSDMD cleavage with EC_50_ values of around 5 µ*M*. Structural characterization of the GSDMD-CTD–Peptide 2 complex suggests that the peptide recognizes the same binding pocket as the caspase. Although the peptide has linearized in our complex structure, the β-hairpin conformation from the two linearized peptides in the crystallographic asymmetric unit is required to fully occupy the exosite. Our structure indicates that fully occupying the exosite with cyclic peptides or small molecules provides a unique starting opportunity to inhibit caspase binding to hGSDMD. This aligns with the results of our hGSDMD-cleavage TR-FRET assay, which indicate that the cyclic feature is required for inhibition.

The hGSDMD-CTD–Peptide 2 complex structure reveals that the conserved GSDMD exosite possesses inherent flexibility and is likely to accommodate a variety of substrates. The hydrophobic exosite features a lining of charged residues, which are likely to play critical roles in substrate binding. Previous studies indicated that the active site is not involved in substrate recruitment and would likely be disordered in the reported full-length apo hGSDMD structure (PDB entry 6n9o; Liu *et al.*, 2020 ▸). However, the flexible active-site linker region rigidifies after binding the substrate caspase-1 (PDB entry 6vie). Unfortunately, the active-site sequence (272-FLTD-275) is not involved in our construct (residues 276–482) to elucidate potential conformational changes upon peptide binding. The full length hGSDMD–peptide complex structure would provide further insight into the ability to design tools to block the enzymatic activity.

Our high-resolution co-crystal structure of hGSDMD-CTD in complex with Peptide 2 provides a structural framework for the design of more potent and selective inhibitors, with the structural data correlating well with our TR-FRET GSDMD-cleavage assay results. The structure reveals that the Gly1 residue of Peptide 2 forms a significant hydrogen-bonding interaction with Glu354 of GSDMD and fits well within a hydrophilic pocket (Fig. 4 ▸). Consequently, substituting Gly1 with a bulky hydrophobic phenylalanine (Peptide 3) is likely to disrupt this interaction, which is reflected in a threefold to fourfold decrease in potency in the TR-FRET cleavage assay (Table 2 ▸). Peptide 4, containing a P3–P5 change (KKI to IKK), showed increased potency in both cleavage assays, suggesting that the P3–P5 change may increase nonspecific binding or that the IKK substitution compensates for binding energy lost because of the G1F mutation. Ultimately, this structure serves as a reliable model to guide the future design of cyclic peptides and small molecules. Although peptide inhibitors present druggability challenges due to poor membrane permeability, our hGSDMD–Peptide 2 complex structure has identified a minimal pharmacophore for substrate binding at the exosite. The detailed molecular interactions and potential hydrogen-bonding network with the surrounding negatively charged residue network could provide opportunities to guide further iterative design strategies and could be utilized as the basis for a small-molecule virtual screen.

## Supplementary Material

PDB reference: gasdermin D bound to a peptide, 9e0v

Supplementary Figures. DOI: 10.1107/S205979832600344X/ai5017sup1.pdf

## Figures and Tables

**Table 1:** Amino-acid sequences for the designed Peptides 1–4 6CLW, 6-chlorotryptophan.

Compound	N-terminus	P1	P2	P3	P4	P5	P6	P7	P8	P9	P10	P11	P12	C-terminus
Peptide 1	H_2_N–	G	C	I	K	K	A	V	W	F	K	C	G	Amide
Peptide 2	H_2_N–	G	C	I	K	K	A	V	6CLW	F	K	C	G	Amide
Peptide 3	H_2_N–	F	C	I	K	K	A	V	W	F	K	C	G	Amide
Peptide 4	H_2_N–	F	C	K	K	I	A	V	6CLW	F	K	C	G	Amide

**Table 2:** Biochemical characterization of peptides in GSDMD-cleavage TR-FRET and caspase-1 activity assays GSDMD-cleavage TR-FRET and caspase-1 activity assays were performed as described in Section 2. EC_50_ values are reported as the geometric mean of three independent experiments (*n* = 3) with the range of individual values in parentheses.

Compound	GSDMD-cleavage TR-FRET assay, EC_50_ (µ*M*)	Caspase-1 activity assay, EC_50_ (µ*M*)	Selectivity†
Peptide 1	4.8 (3.7–6.8)	>100	>20×
Peptide 2	5.3 (4.4–6.4)	83 (78–87)	16×
Peptide 3	19 (17–24)	93 (86–99)	5×
Peptide 4	2.5 (1.9–3.2)	22 (17–26)	9×

† Selective inhibition of GSDMD cleavage compared with nonspecific inhibition of caspase-1 activity was calculated as the ratio of GM EC_50_ values (caspase-1 GM EC_50_/GSDMD cleavage GM EC_50_)

**Table 3:** X-ray diffraction data-collection and refinement statistics Values in parentheses are for the highest resolution shell.

Data collection	
Space group	*P*4_3_2_1_2
*a*, *b*, *c* (Å)	47.57, 47.57, 194.72
α, β, γ (°)	90.00, 90.00, 90.00
Resolution (Å)	48.68–1.63
No. of reflections (total/unique)	306744/26002
*R*_meas_† (%)	0.053 (0.676)
〈*I*/σ(*I*)〉	25.9 (2.1)
CC_1/2_‡ (%)	99.9 (70.1)
Completeness (%)	89.3 (50.7)
Multiplicity	11.8 (6.9)
Refinement	
*R*_work_/*R*_free_§	0.21/0.23
CC_work_/CC_free_	0.94/0.94
No. of atoms	
Protein	1580
Heterogen	15
Water	166
*B* factors (overall mean)	26.24
R.m.s. deviations	
Bond lengths (Å)	0.008
Bond angles (°)	0.94
Ramachandran plot, favored/disallowed (%)	99.01/0.49
PDB code	9e0v

† *R*_meas_ = 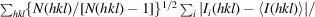

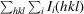
, where *I*_*i*_(*hkl*) and 〈*I*(*hkl*)〉 are the *i*th and mean measurement of the intensity of reflection *hkl* and *N*(*hkl*) is the multiplicity.

‡ This is the Pearson’s correlation coefficient of randomly split half datasets (Karplus & Diederichs, 2012). CC_1/2_ = 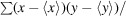

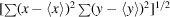
, where *x* and *y* are randomly split half datasets.

§ *R*_work_ = 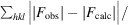

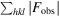
, where *F*_obs_ and *F*_calc_ are the observed and calculated structure factors, respectively; *R*_free_ is the *R* value obtained for a test set of reflections consisting of a randomly selected 5% subset of the data set that was excluded from refinement.
