# Supplementary material for: Peptide binding at the gasdermin D exosite reveals the structural basis for targeting the site
Source: Acta Crystallogr D Struct Biol. 2026 May 13;82(Pt 6):615–25. doi: 10.1107/S205979832600344X (PMC13224929; doi:10.1107/S205979832600344X)
Supplement: Supplementary file 1 [file d-82-00615-sup1.pdf]

STRUCTURAL BIOLOGY

Volume 82 (2026)

Supporting information for article:

Peptide binding at the gasdermin D exosite reveals the structural basis for targeting the site

Renjing Wang, Thu Ho, Aimie Ogawa, Kartika Widjaja, Zachary Brown, Song Yang, Pei Huo, Michael C. Gregory, Aman Singh Singh, Senthil Perumal, Songnian Lin, Alan C. Cheng, Lindsay S. Garrenton, James Mu, Anthony Ogawa and Jill E. Chrencik

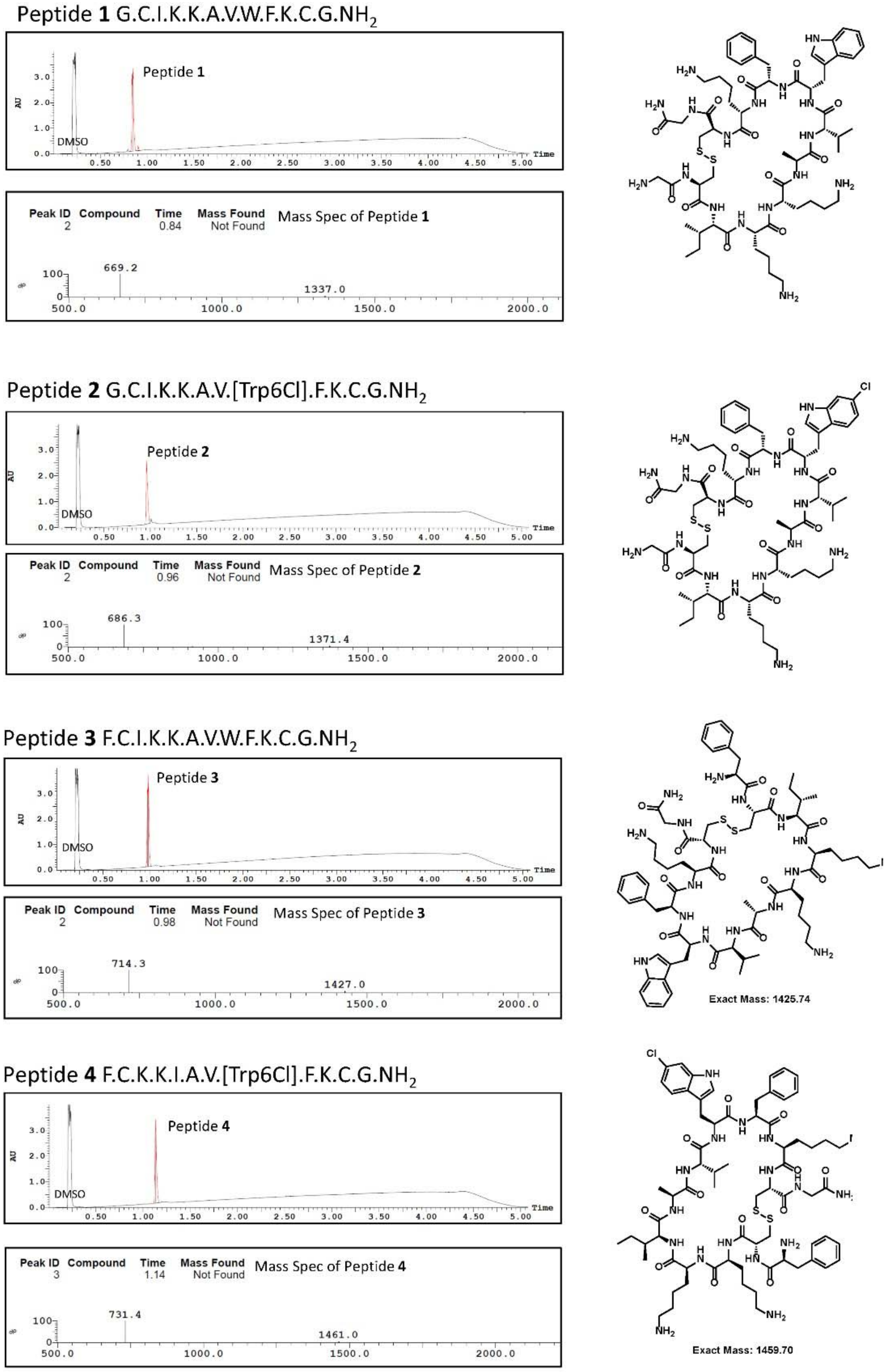

Figure S1 Mass spectrometry characterization of Peptide 1-4.

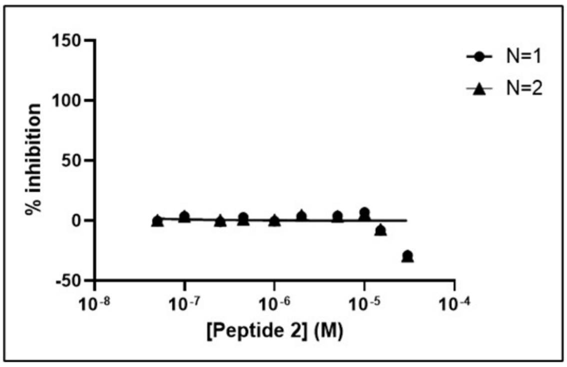

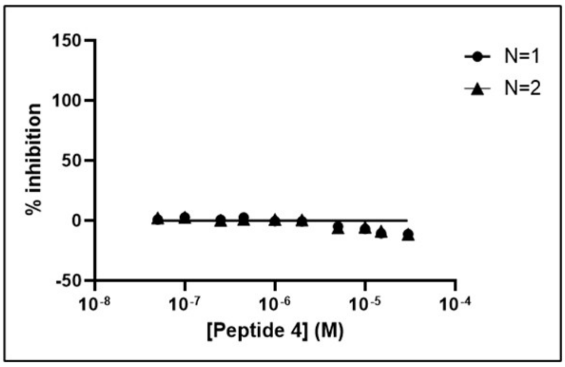

Figure S2 Biochemical characterization of exosite peptides in GSDMD cleavage assay in reducing conditions. GSDMD cleavage TR-FRET assays were performed in the presence of increasing concentrations of peptides up to 30 µM as described in Materials and Methods but with 2 mM DTT included in the assay buffer. Data for two independent experiments (N=1, black circles and N=2, black triangles) are plotted.

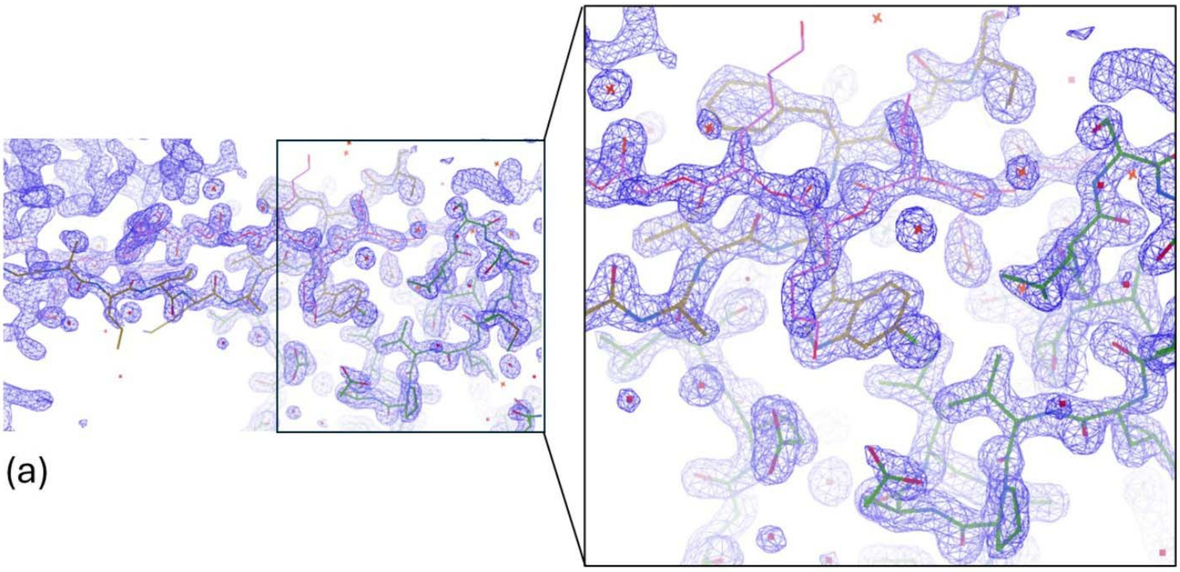

(a)

(b)

Figure S3 Electron density maps of the peptide-binding pocket. (a) Electron density map of the peptide-binding pocket and the symmetry-related pocket, showing the β-hairpin formed by two linearized peptides in the asymmetric unit. (b) Close-up view of the electron density around Peptide 2 in its binding pocket: hGSDMD-CTD is shown in green, Peptide 2 in brown, and the symmetry-related Peptide 2 in purple.

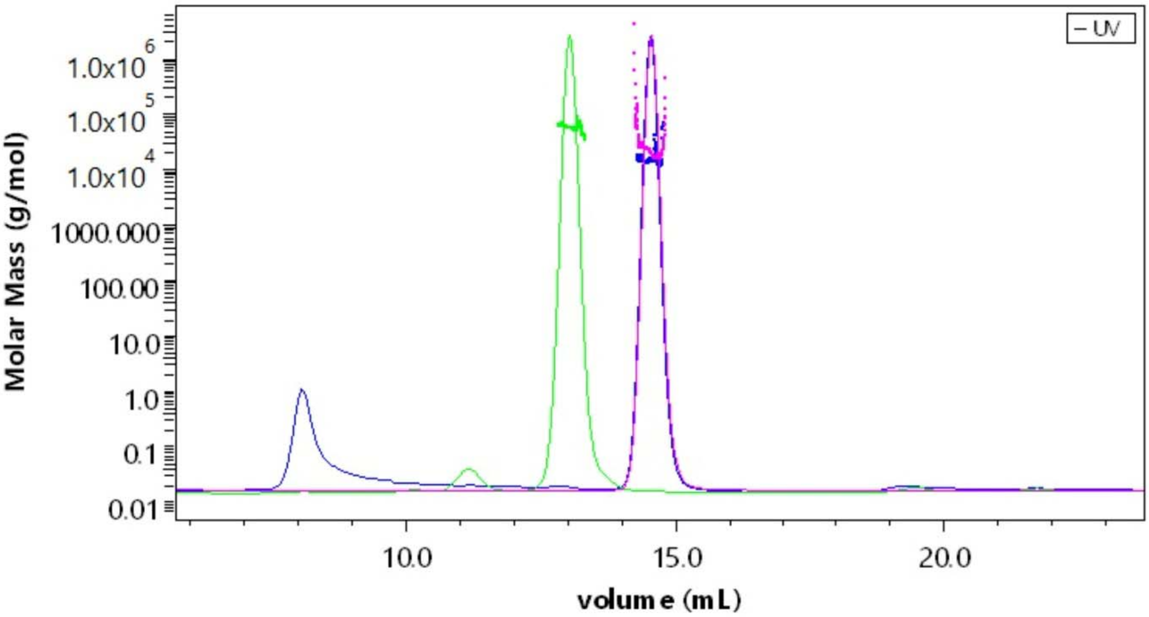

Figure S4 SEC-MALS show that hGSDMD-CTD remains a monomer in the presence and absence of peptide. SEC-MALS of hGSDMD-CTD samples were run on a S200 10/300 column. Both Apo and Peptide 2 bound hGSDMD-CTD are monomeric with observed MW of 2.50 x 10^4 (+/- 15.86%) and 1.74x 10^4 (+/- 23.57%) respectively.

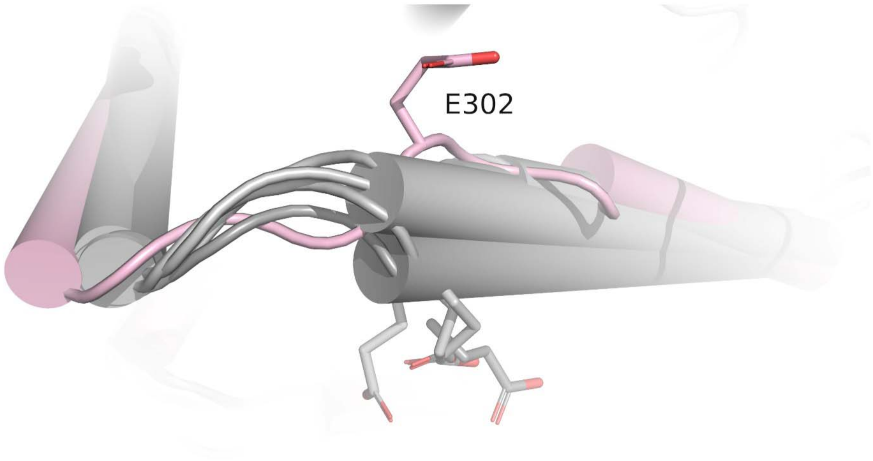

Figure S5 Superposition of hGSDMD-CTD/Peptide2 region (residue 300-311) colored in pick with 4 published structures in grey (related to Fig. 3 and 4). Full length apo hGSDMD (PDB: 6N9O) with RMSD 2.170, apo hGSDMD-CTD (PDB: 5NH1) with RMSD 2.137, Full length mGSDMD/Caspase 1 complex (PDB: 5NH1) with RMSD 1.763 and hGSDMD-CTD/Caspase 1 complex (PDB: 6KN0) with RMSD 1.602.

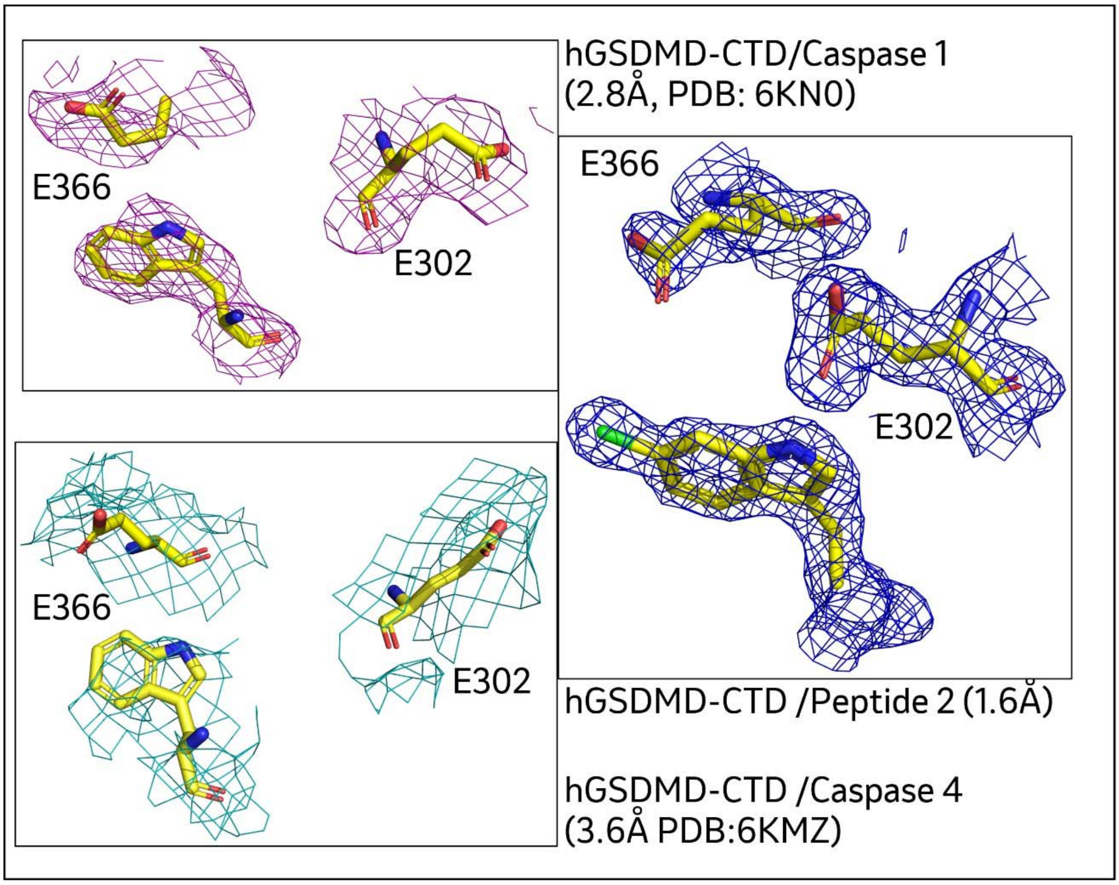

Figure S6 Electron density maps comparison of the key interaction with Tryptophan (related to Fig. 4) The electron density comparison of the residue E366 and E302, which involved in forming the hydrogen bond with Tryptophan of Caspase and peptide, respectively. The top left panel is for hGSDMD-CTD/Capase 1 with resolution 2.8Å (PDB: 6KN0), the bottom left panel is for hGSDMD-CTD /Caspase 4 with resolution 3.6Å (PDB: 6KMZ), and the right panel is for hGSDMD-CTD /Peptide 2 with resolution 1.6Å.

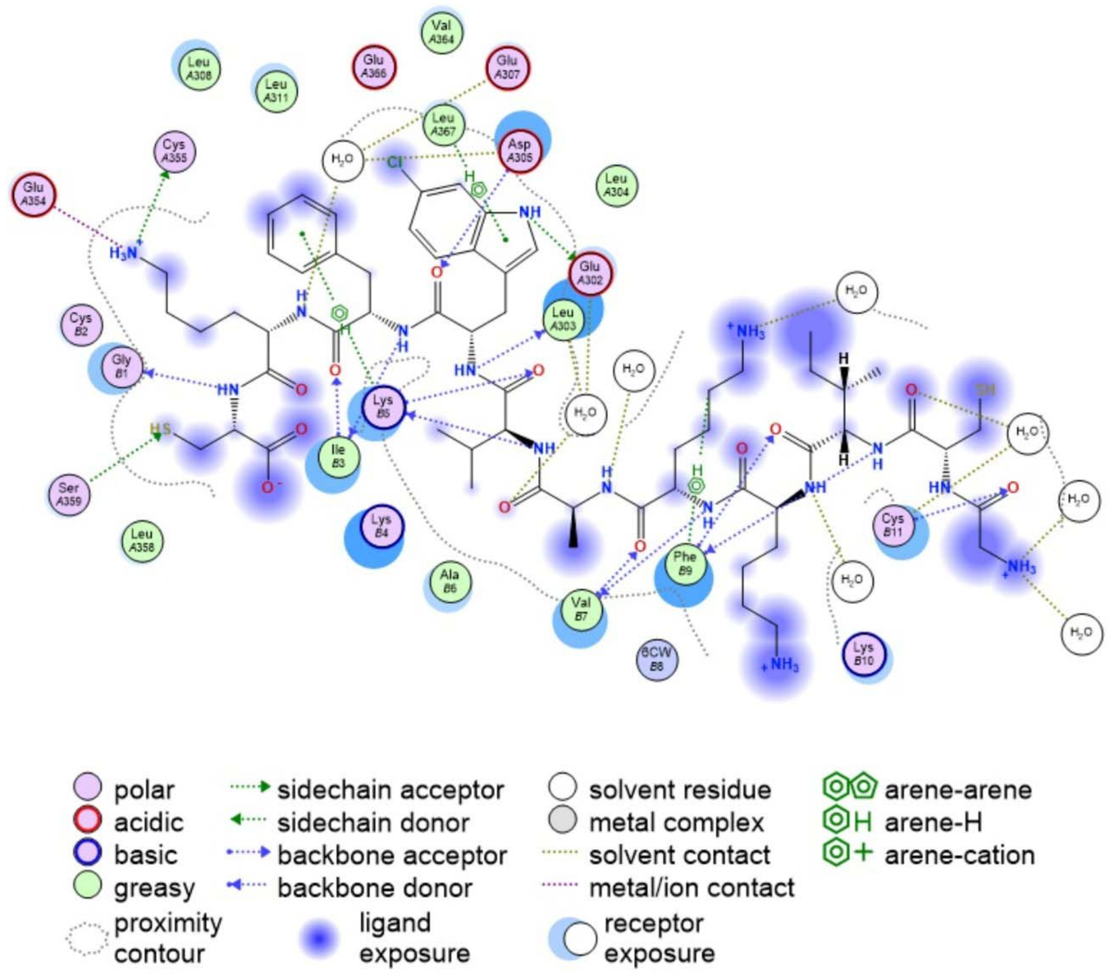

Figure S7 hGSDMD-peptide interaction diagram (related to Fig. 4) 2D display of the hGSDMD-Peptide 2 interactions. Peptide 2 β-sheets intern chain interactions are also indicated. Peptide 2 is centered. hGSDMD are labeled as A with residues and the other peptide are labeled as B with residues.
